# Medical and Physical Intelligence

**Published:** 1806-11

**Authors:** 


### MEDICAL AND PHYSICAL INTELLIGENCE.

### Royal Jennerian Society for the Extermination of the Small-Pox.

*Salisbury Square, Oct.* 14, 1806.

A Meeting of the Medical Council was held at the Central House, on Thursday the 2d of October, for the purpose of electing a Resident Inoculator and Medical Secretary, in place of John Walker, M. D. who resigned that office the 8th of August last. The Council, on this occasion, elected Doctor James Sheridan Knowles, a gentleman of distinguished talents, and a zealous promoter of Vaccination. Dr. K. had been, for some time before his election, engaged in the task of representing, by a series of engravings, the vaccine vesicle, and the deviations from its genuine form, which take place under a variety of circumstances. These representations were examined and highly approved by Dr. Jenner; some of them have been engraved, and appear in Dr. Willan's Treatise on Vaccine Inoculation.—The Correspondents of the Royal Jennerian Society will therefore, in future, address their applications for vaccine fluid, or for other medical purposes, to Dr. Knowles, No. 14, Salisbury-square.——As no exertion or expence has been spared by the Directors and Medical Council, in their endeavours to diffuse the benefits of the Vaccine Inoculation, and to accommodate practitioners in every part of the kingdom, they hope those gentlemen will communicate, through the Secretary, whatever may occur deserving of attention, and that they will co-operate in giving effectual support to the Society founded by Dr. Jenner on principles, the result of which must prove not less honourable to himself than beneficial to the nation.

N. B. The privilege of receiving or sending letters post-free has been withdrawn from the Society.

---

At a meeting of the Academy of useful Sciences at Erfurt, Mr. Buchner read a memoir on inoculation for the natural small-pox, and on the result of the first vaccination at Bergen in Norway. He gives a circumstantial account of the latter, and states a remarkable case which fell under his observation in the performance of his medical duty. He was sent for to a child a year old, belonging to Capt. Paasche, who commanded a ship, and was absent at the time on a voyage to France. The mother imagined that the symptoms of the disorder proceeded from dentition; but Mr. Buchner soon discovered all those that usually attend the natural small-pox. Before its eruption, he several times endeavoured, but in vain, to prevail on the mother to have her other two children vaccinated. The next day the eruption appeared, the small-pox small-pox became malignant, and on the sixth day the child died. The disconsolate mother then repaired to the physician, imploring him to save her two remaining children. He resolved to vaccinate them, after a suitable preparation. He directed them both to be removed to the most distant apartment in the house, to be put into a warm bath, to be well rubbed, and all the clothes they had before worn to be changed. The vaccination was successful; the punctures became inflamed, the eruption took place at the proper time, and the tumours approached to perfect maturity. But after the eighth day, the two children had a very restless night; they felt an inclination to vomit, had head-ach; in short, all the symptoms which usually precede the natural small-pox. The next day the eruption actually took place, and the bodies of the two children were covered with it. This small-pox was neither of the favourable nor yet of the malignant kind, and both the children got well over the crisis. But it was remarkable, that the vaccine-pocks continued their progress, and their scabs did not fall off till after the desiccation of those of the small-pox.

---

Mr. Gardner, of the City Dispensary, has, in the course of some galvanic experiments, been led to try the effect of the galvanic fluid upon vegetable infusions. Turmeric with distilled water was first submitted to trial; the circuit being made with iron wires, gas was given out from both, and the infusion became gradually changed from a bright yellow to a deep brown, beginning at the upper part of the tube, both wires became black, probably from the oxygen evolved from the water. The same quantity of the infusion of litmus was then subjected to the galvanic action; in a few minutes the blue tinge began so fade; the liquor became diaphanous, and at length exhibited a greenish colour, gas being given out from bosh wires, which were also turned black. From these experiments, he conceived an alkali had been formed during the operations; to prove the truth of the conjecture, he was enabled to restore the blue colour to the litmus, by means of dilute sulphuric acid. He repeated the experiment several times with the same success. Syrup of violets, diluted with an equal quantity of distilled water, and galvanized with silver and iron wires, turned as perfectly green as it could have done on the addition of pure ammonia, potash, or soda.

---

Dr. Spalding, of Portsmouth, New Hampshire, complains that not a single volume of this Journal, later than the 13th, is to be found in America, and ascribes the circumstance to some inattention on the part of our Publisher. On enquiry we find, however, that the bad faith and the bad credit of the American booksellers, are the causes why very few good books are exported from England to the United States. The merchants are not paid for one half they send, consequently they seldom export any books for

America,

America, except such as they can purchase at the price of waste paper. Our publisher says he sent quantities of this Journal to New York, with little or no return, for several years; and that he has not yet received from his Agents a tenth of the sum due to him. The complaint of Dr. SPALDING has frequently been made to us by other American Correspondents, and we embrace this opportunity of explaining the origin of the evil, conceiving that it may be easily removed by the establishment and support of some Bookselling House in America, of unquestionable punctuality and credit.

---

Mr. MILBURNE's Autumnal Course of Lectures on Anatomy, Physiology, and Operations of Surgery, will recommence on the 10th instant. Printed particulars may be had of Mr. Milburne, at his house, No. 31, St. James's Street.

---

Mr. BARCLAY's new work on the Muscles is expected shortly.

Mr. BURNS, of Glasgow, has a practical work on Hæmorrhage in the press.

Dr. COGAN is preparing for the press an Ethical Treatise on the Passions, founded on the principles advanced in the Philosophical Treatise. The first part, which is expected to appear in the ensuing winter, will consist of three Disquisitions; on the agency of the passions in the pursuit of well-being; on the intellectual powers, as directories in the pursuit; and on the nature and sources of that well-being, of which the human species is susceptible.

Mr. VETCH is preparing a work on Ophthalmia.

---

